# Re‐exploring tissue regeneration by novel spatial transcriptomics technologies

**DOI:** 10.1002/ctm2.1127

**Published:** 2022-11-24

**Authors:** Xiaoyu Wei, Hanbo Li, Jiaxin Du, Xiaoqi Zeng, Xun Xu, Liang Chen, Ying Gu

**Affiliations:** ^1^ BGI‐Hangzhou Hangzhou China; ^2^ BGI‐Shenzhen Shenzhen China; ^3^ BGI‐Qingdao Qingdao China; ^4^ Lars Bolund Institute of Regenerative Medicine Qingdao‐Europe Advanced Institute for Life Sciences BGI‐Qingdao Qingdao China; ^5^ College of Life Sciences University of Chinese Academy of Sciences Beijing China; ^6^ Guangdong Provincial Key Laboratory of Genome Read and Write BGI‐Shenzhen Shenzhen China; ^7^ Hubei Key Laboratory of Cell Homeostasis RNA Institute College of Life Sciences Wuhan University Wuhan China

1

One of the ultimate goals of regenerative medicine is to replace damaged tissues using natural growth and repair mechanisms, which barely remain in adult human body. In contrast, certain lower vertebrates, such as salamanders and zebrafish, exhibit an incredible ability to regenerate various damaged body parts both structurally and functionally. These animals with such natural gifts have therefore drawn the long‐term attention of biologists, who are dedicated to uncovering the molecular mechanisms that direct programmed regeneration and how regenerative medicine practice would learn from fundamental scientific discoveries. While early efforts focused on one or a few molecules/pathways/cell types have extensively substantiated our understanding in this field,^1^ the gap between the enormous complexity of regenerative responses and the low‐throughput readout of the canonical methods has hindered scientists from elaborating the mechanism systematically.

To tackle this bottleneck issue, single‐cell RNA sequencing technologies (scRNA‐seq) were introduced into the field recently. They can capture molecular characteristics and dynamics of every known cell type and help identify new or transient cell states. Applying scRNA‐seq to axolotl limb regeneration, researchers discovered a critical cell state transition from connective tissue to blastema cells required for lost tissue reconstruction in a later stage,^2^ possibly via the epithelial‐mesenchymal transition mechanism.^3^ ATAC‐seq data further provided rich chromatin regulatory information and deepened our understanding of the molecular basis of limb regeneration dynamics.^4^

Single‐cell multi‐omics have remarkably empowered us with global pictures and networking perspectives, yet they have inherent limitations, too. Spatial information of cells is lost when constructing scRNA‐seq libraries, including cell distribution and identities of the neighboring microenvironment, thus hampering the in‐depth investigation of the biological process of a tissue or organ in situ. To this end, new spatial transcriptomics technologies developed recently stand out to be a solution, such as microdissection‐based cell capture and sequencing laser capture microdissection (LCM, Geo‐seq), high throughput in situ hybridization (smFISH, MERFISH), in situ sequencing (STARmap), and in situ capturing technologies (Stereo‐seq, Slide‐seq),^5^ some of which (Stereo‐seq, Seq‐Scope, and sci‐Space) reach a single‐cell or subcellular resolution. Combining the accurate information of the transcript coordinate and hundreds of genes detected in a single spot on the chip, scientists can not only obtain the physical location and transcription profile of each cell on the section, but also redefine the cell types and even tissue anatomy of a given organ at the molecular level. Investigations that are difficult for single cell omics method, such as cell state trajectory along the certain anatomical route of tissue for differentiation, and cell‐cell communication and microenvironment within the boundary of disease versus healthy areas, would be achieved by spatial transcriptomics to target key questions in development and disease mechanism.

For example, Chen et al. constructed exquisite spatiotemporal maps of developmental mouse embryos at single‐cell resolution.^6^ Together with the organ atlas of several other species published recently, these spatial transcriptomics profiles provide valuable mechanistic insights into the cell fate specification in developing tissues.^7^ For clinical research, by focusing on the invasive fronts of intrahepatic cholangiocarcinoma, Wu et al. observed the distinct immune microenvironment that may promote tumour invasion.^8^ Ou et al. found unique immune cell types enriched in tumour areas with different metabolic activity.^9^ By analyzing neighboring cell composition from spatial transcriptomics data, Cong et al. identified a specific macrophage subgroup for SARS‐CoV‐2 clearance and inflammation resolution^10^ (Figure 1).

In the regeneration field, a multi‐institute research team led by BGI‐research recently applied Stereo‐seq to reveal how a brain injury heals by itself in axolotls, which was published as the cover story in Science.^11^ By constructing the first single‐cell spatial profile of axolotl telencephalon throughout development, Wei et al. redefined ependymoglial cells (EGCs), the neural stem cells in the axolotl brain, into three transcriptomically distinct subgroups distributed separately in the ventricular zone. During telencephalon regeneration, Stereo‐seq data showed that a new EGC subpopulation from local resident EGCs near the injury site, namely reactive EGCs (reaEGCs), was activated, which proliferated and moved to cover the injury site, then replenished lost neurons through a state transition to intermediate progenitors, immature and eventually mature neurons. Notably, such brain regeneration led by reaEGCs partially recapitulated the developmental program at the molecular level and in the spatial distribution of cells of the same state^11^ (Figure 2A). While scientists have speculated that brain regeneration resembles brain development to some extent, efforts by Wei et al. have demonstrated this long‐standing hypothesis by providing exquisite spatial transcriptomic evidence, which implies that improved activation of progenitor cells might be a promising avenue for brain regeneration. When increasing spatial transcriptomics data of the tissue regeneration in different species become available, cross‐species comparion would be performed to provide more insights into the mechanism of limited regeneration ability of higher vertebrates, and explore possibilities, such as stimulated stem cell activation, to imporve tissue regeneration of mammals, including human, in the future.

**FIGURE 1:**
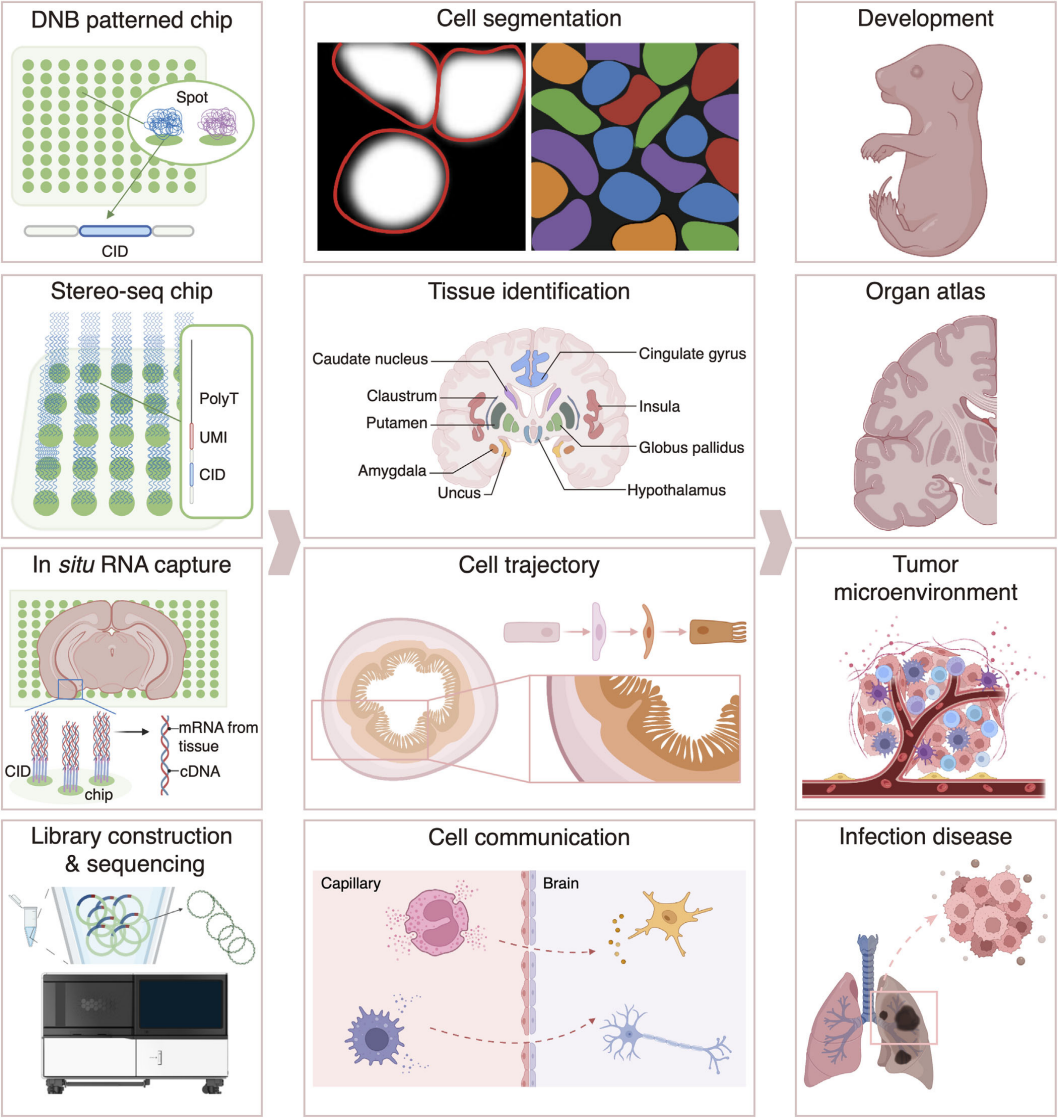
The workflow of Stereo‐seq technology and its application. Cartoons show the DNA nanoball (DNB)‐patterned array chip and the experimental procedure (left), and the analysis strategies of spatial transcriptome data (middle). Spatial transcriptomics technologies have been applied to generate tissue atlases of different species under the physiological or pathological condition for various research directions, for example, embryonic development, tumour microenvironment and infection disease (right).

**FIGURE 2:**
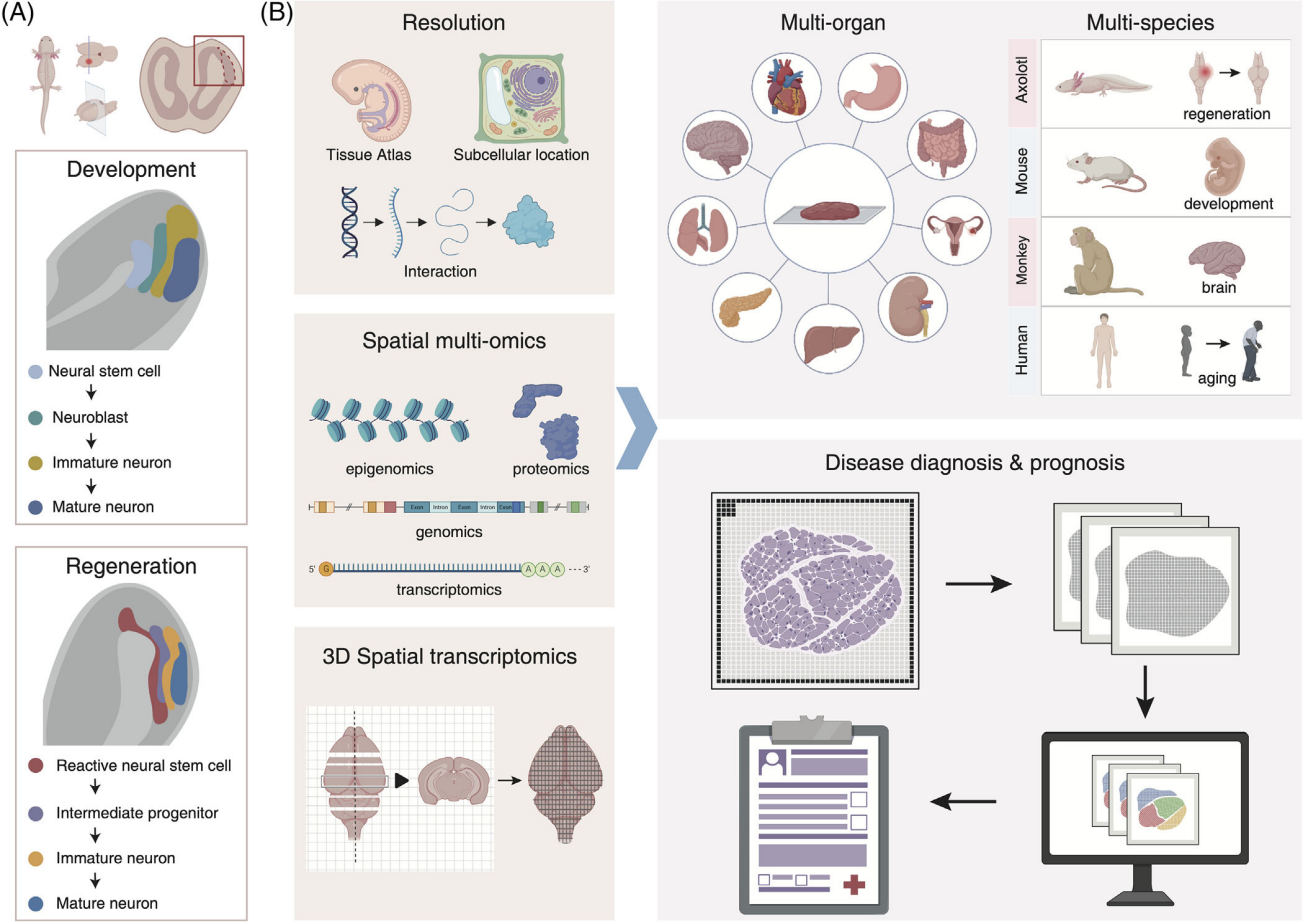
(A) Development and regeneration of the axolotl telencephalon. A neural stem cell subpopulation was activated rapidly to replenish lost neurons in the injury site (bottom) through a process similar to neurogenesis during early development (middle). (B) Future development and applications of spatial transcriptomics technologies include improving resolution to the subcellular level, capturing spatial multi‐omics information and achieving 3D spatial transcriptomics (left). The progress in spatial transcriptomics will contribute to the multi‐organ, multi‐species research in various fields and clinic practice for disease diagnosis and prognosis (right).

Spatial transcriptomics strategies make a glimpse of all the behaviors of all cells in situ at the same time into reality. Here, we anticipate several research directions in spatial‐omics development and potential application in the future. First, technological improvement in sample applicability, higher throughput and transcript capturing efficiency, greater sequencing depth, and more data analyzing tools to expand the application; Second, the development of spatial multi‐omics technologies beyond transcriptomics, including genomics, proteomics, metabolomics, and metagenomics to realize more research perspectives. Third, the accomplishment of spatial transcriptomics from 2D to 3D will provide a complete picture of the research subject at the tissue, cell, and molecular levels. With unprecedented technological advances, spatial‐omics technologies, like Stereo‐seq, will further shift the paradigm of many basic research fields, such as the development of plants and animals, aging, and tissue regeneration. They may also greatly promote our understanding of disease mechanisms from the tissue or even system point of view, such as cancer, infection, and metabolic and neurological diseases. We also anticipate that as spatial‐omics technologies become more available beyond basic research, they may have broader applications in clinical practice, as a comprehensive and advanced dissection tool for disease diagnosis and post‐treatment examination (Figure 2B).

## CONFLICT OF INTEREST

The authors declare no conflict of interest.
